# Challenges in IPC training for non–healthcare workers

**DOI:** 10.1017/ash.2022.124

**Published:** 2022-05-16

**Authors:** Faridah Binte Abdul Majid, Lai Chee Lee, Kwee Yuen Tan, Moi Lin Ling

## Abstract

**Background:** In the last 2 years of the COVID-19 pandemic, Singapore has been forced to explore alternative sites to quarantine persons or manage infected cases during surge periods in a national effort not to overwhelm the public healthcare facilities. External quarantine facilities were created at the EXPO and further extended to D’Resort and other hotels in May 2020. Infection prevention (IP) practices were implemented at these external facilities, where training non–healthcare staff to quickly learn and understand these required practices has been challenging. A team of staff from different clinical disciplines was formed to manage the COVID-19 patients at these facilities. The Infection Prevention and Epidemiology (IPE) department was invited to train all staff, including the clinical team, management agency, and security staff, regarding IP measures. We have described the system and approach used in the rapid training of all staff in IP measures where the goal is zero transmission while providing care to COVID-19 patients. **Methods:** Training materials were developed to facilitate rapid learning by all staff; medical jargon was avoided. Curriculum included precautions to be taken while performing terminal cleaning of patient rooms, serving meals, disinfecting phones and thermometers, as well as donning and doffing personal protective equipment (PPE). “Green” and “red” zones were created to assist staff in remembering appropriate PPE to be used. PPE training was provided using slides and video. Posters were created as a guide for staff at donning and doffing stations. Additionally, the IPE training team utilized an online data collection tool to capture staff completion on IP training and PPE competency for record keeping. We used a ‘soft’ approach because staff members were fearful of the unknown when caring for COVID-19 patients. Daily audits were conducted with immediate concurrent feedback to engage the relevant stakeholders. Infection prevention liaison officers (IPLOs) were appointed to assist in the daily audits. An electronic audit tool was used to facilitate audit and quick analysis. **Conclusions:** The experience gained in the last 2 years has been useful and may provide a template if new external sites are needed in the future because of the potential surge associated with the ο (omicron) variant.

**Funding:** None

**Disclosures:** None

